# Efficacy and safety of milrinone in the treatment of cerebral vasospasm after subarachnoid hemorrhage: a systematic review

**DOI:** 10.5935/0103-507X.20200097

**Published:** 2020

**Authors:** Alex Goes Santos-Teles, Clara Ramalho, João Gabriel Rosa Ramos, Rogério da Hora Passos, André Gobatto, Suzete Farias, Paulo Benígno Pena Batista, Juliana Ribeiro Caldas

**Affiliations:** 1 Critical Care Unit, Hospital São Rafael - Salvador (BA), Brazil.; 2 Escola Bahiana de Medicina e Saúde Pública - Salvador (BA), Brazil.; 3 Universidade de Salvador - Salvador (BA), Brazil.

**Keywords:** Milrinone, Subarachnoid hemorrhage, Vasospasm, intracranial, Milrinona, Hemorragia subaracnóidea, Vasoespasmo intracraniano

## Abstract

**Objective:**

To systematically review the current evidence on the efficacy of milrinone in the treatment of cerebral vasospasm after subarachnoid hemorrhage.

**Methods:**

The Pubmed^®^, Cochrane and Embase databases were screened for articles published from April 2001 to February 2019. Two independent reviewers performed the methodological quality screening and data extraction of the studies.

**Results:**

Twenty-two studies were found to be relevant, and only one of these was a randomized control trial. Studies showed marked heterogeneity and weaknesses in key methodological criteria. Most patients presented with moderate to severe vasospasm. Angiography was the main method of diagnosing vasospasm. Intra-arterial administration of milrinone was performed in three studies, intravenous administration was performed in nine studies, and both routes of administration in six studies; the intrathecal route was used in two studies, the cisternal route in one study and endovascular administration in one study. The side effects of milrinone were described in six studies. Twenty-one studies indicated resolution of vasospasm.

**Conclusion:**

The current evidence indicates that milrinone may have a role in treatment of vasospasm after aneurysmal subarachnoid hemorrhage. However, only one randomized control trial was performed, with a low quality level. Our findings indicate the need for future randomized control trials with patient-centered outcomes to provide definitive recommendations.

## Abstract (pt)

**Objetivo:**

Revisar sistematicamente a evidência atual da eficácia de milrinona no tratamento do vasoespasmo cerebral após hemorragia subaracnóidea.

**Métodos:**

Triaram-se as bases de dados Pubmed®, Cochrane e Embase quanto a artigos publicados entre abril de 2001 e fevereiro de 2019. Dois revisores independentes realizaram uma triagem metodológica da qualidade e a extração dos dados dos estudos.

**Resultados:**

Encontraram-se 22 estudos considerados relevantes, sendo que apenas um deles era um ensaio randomizado controlado. Os estudos demonstraram acentuada heterogeneidade e debilidade de seus critérios metodológicos. A maioria dos pacientes apresentava vasoespasmo moderado a grave. O principal método para diagnóstico do vasoespasmo foi a angiografia. Em três estudos, realizou-se administração de milrinona por via intra-arterial; em nove estudos, a administração foi endovenosa, e, em seis estudos, utilizaram-se ambas as vias de administração. A via intratecal foi utilizada em dois estudos, em um estudo, a administração foi realizada via cisterna e, em um estudo, a via de administração foi a endovascular. Os efeitos colaterais de milrinona foram descritos em seis estudos. Vinte e um estudos indicaram a resolução do vasoespasmo.

**Conclusão:**

A evidência atual indica que o uso de milrinona teve um papel no tratamento do vasoespasmo após hemorragia subaracnóidea aneurismática. Contudo, só foi realizado um ensaio randomizado controlado, com baixo nível de qualidade. Nossos achados indicam a necessidade de futuros estudos randomizados controlados com desfechos centrados no paciente, com o fim de proporcionar recomendações definitivas.

## INTRODUCTION

Aneurysmal subarachnoid hemorrhage (aSAH) displays high mortality and morbidity rates despite modern neurosurgical techniques, new imaging modalities, and improved processes for the care of such patients.^(1)^ Aneurysmal subarachnoid hemorrhage is associated with systemic conditions such as cardiogenic shock, injuries to gut motility and pulmonary edema.^(2-4)^ Hydrocephalus, edema, intracranial hypertension and delayed cerebral ischemia (DCI) resulting from vasospasm are common and serious complications after subarachnoid hemorrhage.^(5,6)^ The mortality rate is near 50%, and only 14% of patients survive without sequelae.^(1,5)^

Vasospasm is the most significant complication leading to increased mortality and morbidity after the initial event in patients suffering from aSAH^(7)^ and remains the main factor associated with DCI.^(5,7,8)^ This condition usually starts between the fourth and twelfth day and appears in approximately 70% of the patients who survive the first day after aSAH.^(9,10)^

Cerebral vasospasm is associated with complex mechanisms such as blood-brain barrier disruption, microthrombosis, cortical spreading depolarization and loss of cerebral autoregulation.^(11,12)^ However, the exact physiological pathways are still unclear, and thus, it is difficult to define the best treatment for this condition.^(13)^

A large number of studies have been performed to establish a safe and effective treatment for vasospasm.^(9,12,14)^ In addition to continuous neurological monitoring, the use of nimodipine, associated with euvolemia and hypertension, remains the standard therapy for prevention of DCI.^(12)^ Although the exact mechanism of nimodipine is unclear, this drug improves neurological outcomes and contributes reduction in mortality rates.^(15)^

Since 2001, the use of milrinone has been reported for treatment of vasospasm.^(16)^ Milrinone is a phosphodiesterase inhibitor drug, a noncatecholamine, nonglycosidic vasodilator with a positive inotropic effect.^(17)^ The role of milrinone in the treatment of vasospasm has been described in retrospective studies and many case reports. However, the safety and efficacy of this therapy remain unclear.

In this study, we systematically reviewed the literature to assess the efficacy and safety of milrinone administration for treatment of cerebral vasospasm after subarachnoid hemorrhage.

## METHODS

An electronic search was performed in the Pubmed, ^®^ Cochrane and Excerpta Medica dataBASE (EMBASE) databases without restriction on publication year. The search was performed in August 2019 and included studies from April 2001 to February 2019. The following search strategy was used: ("Vasospasm" [MeSH term] OR "Intracranial Vasospasm" OR "Intracranial Vasospasms" OR "Intracranial Vascular Spasm" OR "Intracranial Vascular Spasms" OR "Intracranial Angiospasm" OR "Intracranial Angiospasms" OR "Cerebral Vasospasm" OR "Cerebral Vasospasms" OR "Cerebrovascular Spasm" OR "Cerebrovascular Spasms" OR "Cerebral Angiospasm" OR "Cerebral Angiospasms" OR "Cerebral Artery Spasm" OR "Cerebral Artery Spasms") AND ("Subarachnoid Hemorrhage" [MeSH term] OR "Subarachnoid Hemorrhages" OR "Aneurysmal Subarachnoid Hemorrhage" OR "Aneurysmal Subarachnoid Hemorrhages" OR "Spontaneous Subarachnoid Hemorrhage" OR "Spontaneous Subarachnoid Hemorrhages" OR "Perinatal Subarachnoid Hemorrhage" OR "Perinatal Subarachnoid Hemorrhages" OR "Intracranial Subarachnoid Hemorrhage" OR "Intracranial Subarachnoid Hemorrhages") AND ("Milrinone" [MeSH term] OR "Win-47203" OR "Win 47203" OR "Win47203" OR "Primacor" OR "Corotrope" OR "Corotrop" OR "Lactate").

Studies were included if they were published in the English language, with human subjects, including patients affected by subarachnoid hemorrhage (traumatic or aneurysmal), patients treated with milrinone (arterial, venous, cisternal or intrathecal administration), and without restrictions on age, sex, severity of the vasospasm and publication year. Studies with animals and review studies were excluded.

Data extraction was performed using State of the Art through Systematic Review (StArt).^(18)^ All papers were reviewed by two independent authors, and conflicts were resolved by consensus among the authors. The Preferred Reporting Items for Systematic Reviews and Meta- Analyses (PRISMA) checklist was used to improve the reporting of this systematic review.^(19)^ The quality of evidence was assessed by the Grading of Recommendations, Assessment, Development and Evaluation (GRADE).^(20)^ The overall confidence was classified as high, moderate or low Evidence Level^(20,21)^ (Appendix 1).

The following data were extracted: year of publication, number of centers, study design, sex of the patient, age, Fisher scale, Hunt & Hess scale, vasospasm grade, vasospasm cause, aneurysm localization, number of patients, routes of administration (venous or arterial), dose, adverse effects, diagnostic tool, screening tool and efficacy.

Considering the heterogeneity of the studies, meta-analysis could not be performed, and a descriptive systematic review was conducted.

## RESULTS

### Study selection

**Figure 1:**
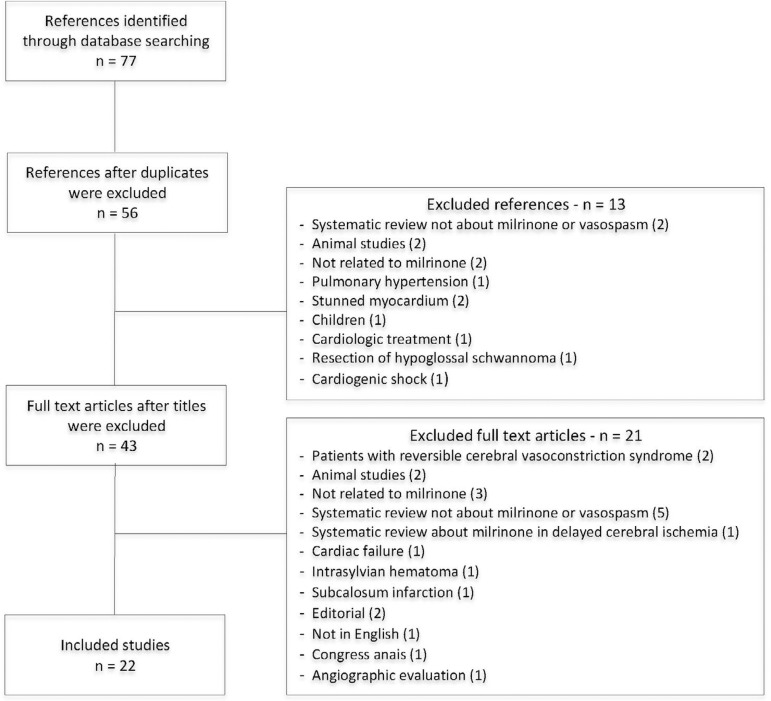
Diagram of the study search and selection processes.

Details on the search and selection processes can be found in figure 1. Overall, 77 articles were retrieved, and 55 were excluded for several reasons, leaving 22 articles eligible to the systematic review.

Of the 22 studies eligible for review,^(1,5,14,16,22-39)^ six illustrated non-controlled observational retrospective studies,^(14,30,32-35)^ 14 were non-controlled observational prospective studies,^(1,5,16,22-29,36-38)^ one was a controlled observational retrospective study^(31)^ and only one was a randomized controlled trial (RCT).^(39)^ All were single-center studies.

### Study characteristics

Characteristics of the studies are listed in table 1. The sample size ranged from 1 to 142 patients. Overall, 641 patients were evaluated in all studies, 387 (60.3%) of which were female, and the average age was 52.4 (23 - 82). Nineteen studies were performed on patients with aSAH,^(1,5,14,16,23,24,25,27-37,39)^ one study included a patient with pretuncal subarachnoid hemorrhage,^(22)^ one study evaluated patients with traumatic subarachnoid hemorrhage^(26)^ and one study did not describe the subarachnoid hemorrhage etiology.^(38)^ The aneurysmal location varied among all studies (Table 1). In most studies, the Fisher scale and Hunt & Hess scale were used to evaluate patients. Subarachnoid hemorrhage grade by the Fisher scale was described in 19 studies,^(1,5,14,16,22-29,31,33,34,36-39)^ and 18 of those studies^(1,5,14,16,23-29,31,33,34,36-39)^ included patients presenting moderate to severe subarachnoid hemorrhage (Fisher 3 - 4).

**Table 1:** Characteristics of identified publications examining the efficacy and safety of milrinone in cerebral vasospasm after subarachnoid hemorrhage.

Study	Design	n	Age mean (SD)	Female sex n (%)	Fisher	Hunt & Hess	Etiology	Aneurysm location	Route	Dosage	Screening method	Confirmatory method	Main results
Romero et al.^(1)^	Observational, uncontrolled, prospective	8	50 ± 10	1 (12.5)	3 - 4	NA	Aneurysmal	Pcom: 3 Acom: 2 MCA: 2 ICA: 1	IA	Infusion 0.25mg/minute Maximal 15mg	TCD	Angiography	All patients had significant angiographic response (> 20% decrease in stenosis) after milrinone
Fraticelli et al.^(5)^	Observational, uncontrolled, prospective	22	45 ± 11	15 (68.1)	2 - 4	NA	Aneurysmal	Pcom: MCA: 5 BA: 2 Acom: 7 ACA: 2 ICA: 1 Not available: 1	IA/IV	Infusion 8mg IA and 0.5-1.5mcg/kg/minute IV	TCD	Angiography	Intra-arterial milrinone resulted in a 37% - 53% increase in arterial diameter
Shankar et al.^(14)^	Observational, uncontrolled, retrospective	14	52.7 (31 - 68)	11 (78.5)	3 - 4	1 - 5	Aneurysmal	Acom: 6 MCA: 3 ICA: 2 Pcom: 3	IA	Maximum 1.13mg/kg/day	TCD	Angiography	2/3 of the treated patients had favorable neurologic outcome
Arakawa et al.^(16)^	Observational, uncontrolled, prospective	7	60.6 ± 10.9	4 (57.1)	2 - 3	NA	Aneurysmal	Acom: 5 ICA/Pcom/ MCA: 1 ICA: 1	IA/IV	Infusion 4 - 15mg IA and 0.5 - 0.75mg/kg/minute IV	Angiography or TCD	Angiography and CT	Mean MCA diameter increased from 1.29 ± 0.48mm to 1.83 ± 0.46mm in the M1 segment and from 0.83 ± 0.24mm to 1.49 ± 0.25mm in the M2 segment
Alturki et al.^(22)^	Observational, uncontrolled, prospective	1	42	1(100)	2	2	Pretuncal	Not aneurysmal	IV	Bolus 5mg and infusion 0.5mcg/kg/minute After 4 days: infusion: 0.25mcg/kg/minute for 24 hours	Angiography	Angiography	The patient was discharged in stable condition without neurological deficit
Anand et al.^(23)^	Observational, uncontrolled, prospective.	1	45	1 (100)	3	NA	Aneurysmal	Acom	IA/IV	3 sessions of 10mg IA followed by 1mcg/kg/minute IV	Angiography	Angiography	The patient recovered fully without neurological deficits
Zeiler et al.^(24)^	Observational, uncontrolled, prospective	1	66	1 (100)	4	4	Aneurysmal	LACA	IV	Infusion 5mg followed by 0.75mcg/kg/minute	Angiography	Angiography	Within minutes of the infusion, the patient resolved the neurologic deficits
Wu et al.^(25)^	Observational, uncontrolled, prospective	1	40	1 (100)	4	3	Aneurysmal	SCA and Pcom	IV	0.75mcg/kg/minute	Angiography	CT	Patient developed cardiomyopathy and arrhythmia
Lasry et al.^(26)^	Observational, uncontrolled, prospective	2	64	1 (50)	3	2 - 3	Traumatic	NA	IV	Bolus 0.1mg/kg and infusion 0.75mcg/kg/minute	NA	Angiography and CT	Improvement of neurologic deficits in both treated patients
Genonceaux et al.^(27)^	Observational, uncontrolled, prospective	1	46	1 (100)	3	3	Aneurysmal	Pcom	IA/IV	Infusion 10mg IA and 0.5mcg/kg/minute IV	TCD	TCD	Consciousness improved after the administration of milrinone
Lannes et al.^(28)^	Observational, uncontrolled, prospective	88	53.4 (34 - 78)	69 (78.4)	1 - 4	1 - 5	Aneurysmal	ACA: 10 Acom: 32 BA: 3 ICA: 2 MCA: 14 Pcom: 22 Other arteries: 5	IV	Bolus 0.1 - 0.2mg/kg. Infusion 0.75 - 1.25mcg/kg/minute	Angiography or TCD	Angiography or TCD	Overall, 48.9% of the patients were able to return to all their previous activities, and 75% had a good outcome. No significant side effects and no medical complications associated with the protocol were observed
Sherif et al.^(29)^	Observational, uncontrolled, prospective	16	NA	NA	2 - 4	1 - 4	Aneurysmal	ACA: 6 ICA: 6 MCA: 2 BA: 1 PICA: 1	EV	4 - 8mg	TCD	TCD	There was angiographic improvement of vessel diameters in 87.5% of patients and an improvement of neurological deficits in 68.5% of patients
Hejčl et al.^(30)^	Observational, uncontrolled, retrospective	34	51 (23 - 73)	27 (79.4)	NA	NA	Aneurysmal	ACA:14 MCA: 13 ICA: 6 BA: 1	IV	Infusion 8mg continued with 0.5 - 1.5mcg/kg/minute for a maximum of 2 weeks	TCD	Angiography	Angiographic results demonstrated decrease in vasospasm in 92% of the patients
Koyanagi et al.^(31)^	Observational, controlled, retrospective	142	62 (51 - 72)	104 (73.2)	4	5	Aneurysmal	ACA: 55 ICA: 39 MCA: 38 PC: 10	IT	0.87mg	Angiography	Angiography	There were fewer DCI events (4% *versus* 14%) in patients treated with intrathecal milrinone compared with those treated without it
Schmidt et al.^(32)^	Observational, uncontrolled, retrospective	73	52 (33 - 82)	50 (68.4)	NA	NA	Aneurysmal	NA	IA	NA	TCD	Angiography	In 91% of cases, there was an improvement in vasospasm. Thirty-day mortality was 11%
Duman et al.^(33)^	Observational, uncontrolled, retrospective	25	59.3 ± 9.8	18 (72)	1 - 4	NA	Aneurysmal	MCA: 32% ICA: 24% ACA: 40% PICA: 4%	IA/IV	Maximal 10 - 16mg milrinone	Angiography	Angiography	In refractory vasospasm, high doses of milrinone led to an improvement in vasospasm
Arakawa et al.^(34)^	Observational, uncontrolled, retrospective	12	64.9 ± 13.8	7 (58.3)	3 - 4	NA	Aneurysmal	BA: 2 Acom: 4 MCA: 2 VA: 1 ICA: 1 ICA/Pcom: 1 BA/SCA: 1	C	Infusion 3.6mg/mL 30mL/hour for 2 weeks	NA	Angiography	Angiographical vasospasm resolved in 9 of the 11 patients who received milrinone administration
Sadamasa et al.^(35)^	Observational, uncontrolled, retrospective	425	NA	NA	NA	NA	Aneurysmal	AC: 492 PC: 78	IT	0.87mg	NA	NA	Delayed ischemic neurological deficit was observed in 16.0% of the patients, DCI was observed in 7.1%, and the overall mortality was 7.2% after milrinone therapy
Santos-Teles et al.^(36)^	Observational, uncontrolled, prospective	1	63	1 (100)	4	5	Aneurysmal	NA	IV	Infusion 0.75mcg/kg/minute IV	TCD	TCD	TCD performed after milrinone administration showed a reduction in cerebral blood flow compatible with the absence of vasospasm
Crespy et al.^(37)^	Observational, uncontrolled, prospective	101	50.5 (42 - 59)	33 (32.6)	3 - 4	NA	Aneurysmal	Acom: 38 MCA: 28 Pcom: 14 ICA: 9 Other: 12	IV/IA	Infusion 8mg IA following by 1mcg/kg/minute IV or only 1mcg/kg/minute IV	TCD	Angiography or CT	The reversion rate was 71% the IA + IV protocol versus 64% in the IV protocol
Katyal et al.^(38)^	Observational, uncontrolled, prospective	1	31	0 (0)	4	NA	Not aneurysmal or traumatic	NA	IV	Bolus 0.1mg/kg IV and 0.5 - 0.75mcg/kg/minute IV	TCD	TCD	TCD performed at 6.5 hours after the initial TCD showed a reduction in mean flow velocities in all vascular territories and cardiac output remained unchanged
Soliman et al.^(39)^	Interventional, randomized, prospective, controlled	90	50.86 ± 9.3	41 (45.5)	2 - 3	NA	Aneurysmal	MCA: 25 ACA: 11 Acom: 32 Pcom: 22	IV	Infusion 0.5mcg/kg/minute IV	TCD	Angiography or CT	The mean cerebral blood flow velocity decreased in the patients of the magnesium group and increased in the patients of the milrinone group. When compared with milrinone magnesium decreased the incidence of cerebral vasospasm

SD - standard deviation; NA - not available; Pcom - posterior communicating artery; Acom - anterior communicating artery; MCA - middle cerebral artery; ICA - internal carotid artery; IA - intra-arterial; TCD - transcranial Doppler; BA - basilar artery;  ACA - anterior cerebral artery; IV - intravenous; CT - computed tomography; M1 - horizontal part; M2 - insular part; LACA - left anterior choroidal artery; SCA - superior cerebellar artery; PICA - posterior inferior cerebellar artery; EV - endovascular; C - cisternal; VA - vertebral artery; AC - anterior circulation; IT - intrathecal; DCI - delayed cerebral ischemia; PC - posterior circulation.

The only RCT included in this review reported a comparison between magnesium and milrinone for treatment of vasospasm,^(39)^ with no placebo group. This study enrolled 90 patients, of which 45 patients received magnesium and 45 patients received milrinone. The results demonstrated that the use of magnesium decreased the incidence of vasospasm compared with the milrinone group.^(39)^ Moreover, in this study, no decrease was noted in the cerebral mean flow velocities in the group treated with milrinone (mean cerebral blood flow velocity before treatment was 88.36 ± 13.75, and mean cerebral blood flow velocity after treatment was 114.71 ± 25.15).^(39)^

### Milrinone administration

Three studies including 95 patients used the intra-arterial route for administration of milrinone,^(1,14,32)^ and nine other studies including 174 patients were conducted using intravenous administration.^(22,25-28,30,36,38,39)^ In six other studies, milrinone was administered by both routes.^(5,16,23,24,33,37)^ Two studies used the intrathecal route,^(31,35)^ one used the cisternal route^(34)^ and the last study did not mention the administration route.^(29)^ One study^(37)^ compared the combination of intra-arterial milrinone infusion followed by intravenous administration versus a continuous intravenous milrinone infusion. The reversion rate of cerebral vasospasm was 71% (59% - 83%) in the intra-arterial + intravenous protocol and 64% (58% - 71%) in the intravenous protocol. This result indicates that continuous IV infusion of milrinone was as efficient as intra-arterial + intravenous infusion for treatment of cerebral vasospasm. The milrinone dosage varied among studies. However, the initial dosage varied between 0.5 to 0.75mcg/kg/minute in most (54%) of the studies (Table 1).

### Vasospasm diagnosis

The screening tool for vasospasm differed from the diagnostic tool in many studies. Patients were screened by serial transcranial Doppler (TCD) in eleven of the studies,^(1,5,14,27,29,30,32,36-39)^ by angiography in five studies,^(20,23,24,33,35)^ by angiography or TCD in two studies^(16,28)^ and by computed tomography (CT) angiography in one study.^(25)^ Different methods were used for diagnosis. Eleven studies used only angiography,^(1,5,14,22-25,30-33)^ two studies used angiography and CT,^(16,26)^ three studies used angiography or TCD^(28,37,39)^ and four other studies used TCD alone to diagnose vasospasm.^(27,29,36,38)^

### Vasospasm resolution

Twenty-one studies demonstrated the resolution of vasospasm after milrinone administration, regardless of milrinone dosage, aneurysm location, vasospasm grade, administration route or methods of diagnosis, as demonstrated in table 1.^(1,5,14,18,22-35)^ On the other hand, the RCT comparing magnesium with milrinone indicated that magnesium was more effective than milrinone in resolution of vasospasm, considering the Glasgow scale and cerebral mean flow velocity.^(39)^

It is important to highlight that vasospasm resolution was defined by angiographic improvement in the diameter of arteries after therapy in 17 studies,^(1,5,14,16,22-25,27,30-35,37,39)^ reduction in cerebral blood flow by TCD in four studies^(26,29,36,38)^ and both methods in one study.^(28)^ Similarly, good outcomes were established considering clinical outcomes and neurological deficits in eight studies.^(22-25,27,32,36,38)^ However, the modified Rankin score was used in five studies,^(14,28,29,35,37)^ the Glasgow coma scale was used in three studies,^(26,30,39)^ the modified Rankin score and Barthel index were used in two studies^(1,33)^ and the World Federation of Neurosurgical Societies (WFNS) grade was used in four studies.^(5,16,31,34)^

### Side effects

Only six studies^(14,25,30,34,37,39)^ reported side effects of milrinone. In one study, intravenous milrinone was associated with cardiomyopathy and arrhythmias in patients without preexisting cardiomyopathy.^(25)^ The other study assessed 12 patients and demonstrated that cisternal administration of milrinone in poor-grade subarachnoid hemorrhage improved the condition of those patients but was associated with pneumocephalus, bacterial meningitis and cerebral infection.^(34)^ The third study described hypotension as a side effect of milrinone.^(14)^ The fourth study with intravenous milrinone, which was a retrospective analysis of 34 patients with aSAH, described hypotension, deterioration of clinical status and temporary hemiparesis as side effects.^(30)^ Crespy et al. described hypokalemia, arrhythmia, increasing dose of norepinephrine and hemodynamical instability regardless of the administration route of milrinone (intra-arterial or intravenous).^(37)^ Finally, in their RCT, Soliman and Zohry reported an increased incidence of hypotension and requirement for dopamine and norepinephrine in the milrinone group compared with patients treated with magnesium sulfate after aSAH.^(39)^

## DISCUSSION

In this systematic review evaluating the use of milrinone in aSAH, the included studies suggested a potential role of milrinone in the treatment of vasospasm after subarachnoid hemorrhage. However, the low quality of the studies and substantial heterogeneity preclude more definitive conclusions.

Approximately 30% of individuals with vasospasm develop DCI. Delayed cerebral ischemia is a serious complication that relies on clinical diagnosis, and as such, it is highly difficult to detect in poor-grade aSAH.^(36,40-42)^ Delayed cerebral ischemia is strongly associated with vasospasm, although other causes may be involved.^(36,43)^

Therapies used for many years in the treatment of vasospasm have now been proven ineffective and potentially harmful.^(40,41,44-48)^ "Triple H" therapy was widely sustained based on its physiological rationale, which seeks to increase cerebral blood flow via induced hypertension, hypervolemia and hemodilution.^(40,45)^ More recent studies demonstrated the damage provoked by this therapy, such as a lower supply of oxygen due to hemodilution, pulmonary edema, myocardial ischemia, hyponatremia, cerebral hemorrhage, and cerebral edema.^(40,45)^ Additionally, statin and magnesium have not shown any potential to eliminate vasospasm.^(49)^ Although hypomagnesemia is common at admission, magnesium was not superior to placebo for reduction of poor outcome after subarachnoid hemorrhage.^(50)^ Nimodipine is currently the most accepted drug for the prevention of DCI. This drug not directly act to reduce vasospasm itself, however nimodipine increases favorable outcomes and decreases mortality.^(12,15)^

Our results indicate that milrinone may have a role as a potential alternative in the treatment of vasospasm, but a lack of evidence exists for its efficacy and safety. Compared with previous therapies, milrinone might represent an option that exerts little impact on volemia, although other adverse effects were described in the studies analyzed in this systematic review. Nonetheless, even in the studies reporting adverse effects, the use of milrinone was associated with resolution of vasospasm.

Only one study did not report an improvement of vasospasm in patients treated with milrinone.^(39)^ This study was a randomized controlled trial that compared milrinone and magnesium as treatments for vasospasm. The results demonstrated that the use of magnesium was more effective than use of milrinone on the resolution of vasospasm. However, a previous larger randomized, double-blinded, placebo-controlled, multicenter phase III trial with patient-centered outcomes concluded that magnesium was not superior to placebo in improving neurologic outcome after aSAH.^(50)^ Because surrogate markers, such as cerebral blood flow velocities, may not be readily translated to patient-centered outcomes, such as mortality or functional outcomes, the presented RCT^(39)^ does not answer the question of whether milrinone is superior to placebo or current medical treatment in the management of aSAH.

A previous systematic review on this subject was published in 2016.^(51)^ Nevertheless, vasospasm continues to present high mortality rates, and new studies have been published in these three years, including the first reported RCT.^(22,25,30,31,33,36-39)^ Thus, this new and updated systematic review may help to inform clinical practice. Although this previous review contemplated different studies inclusion criteria than our study, such as abstracts of conferences and theses, their results are not in disagreement with ours. Furthermore, this review reported the use of milrinone in DCI, and in contrast, this study focused on the occurrence of vasospasm.^(51)^

The systematic review is significant to the literature on aSAH. Although vasospasm is a common neurological event and represents high post-aSAH morbidity, at this moment, no proven effective treatment is available,^(40,41,45,47,48)^ and there is no satisfactory evidence that may aid in decision-making.^(40,41,45,47,48)^ The quantity and quality of the studies demonstrated in this review also elucidates the need for a greater focus and additional research resources on the topic of milrinone as a treatment for vasospasm. We acknowledge that such a study may be difficult to perform, as illustrated by the fact that the only interventional randomized trial registered at clinicaltrials.gov was terminated due to lack of recruitment (NCT02712788).

### Limitations

This review includes limitations. The most important limitation refers to the heterogeneity and the quality of the studies. Almost all studies, except for one RCT, were case reports or case series. Only one randomized controlled trial is available in the literature, and it did not compare milrinone to current standard of care and did not report patient-centered outcomes. It is important to highlight that the drug administration route varied among the studies, and the potential side effects of each one were not described. Due to the heterogeneity of the studies, it was not possible to perform a meta-analysis within the included studies. For instance, the milrinone dosage, drug administration route and diagnostic methods for vasospasm varied within studies, thus limiting the interpretation of data. Although studies with DCI were also included, the evidence with respect to prognosis and the side effects of milrinone in the treatment of vasospasm were described.

## CONCLUSION

Although most of the analyzed studies suggest that milrinone may have a role in the treatment of cerebral vasospasm, the low quality and large heterogeneity in patients, dosing and route of treatment preclude stronger conclusions. Our findings might stimulate future randomized controlled trials with patient-centered outcomes to provide clearer recommendations for clinical practice.

## Appendix 1

**Table:** Assessment of the overall confidence

Study	Evidence level	Estudo	Evidence level
Romero et al.^(1)^	Low	Sherif et al.^(29)^	Low
Fraticelli et al.^(5)^	Moderate	Hejčl et al.^(30)^	Moderate
Shankar et al.^(14)^	Moderate	Koyanagi et al.^(31)^	Moderate
Arakawa et al.^(16)^	Low	Schmidt et al.^(32)^	Moderate
Alturki et al.^(22)^	Low	Duman et al.^(33)^	Moderate
Anand et al.^(23)^	Low	Arakawa et al.^(34)^	Moderate
Zeiler et al.^(24)^	Low	Sadamasa et al.^(35)^	Moderate
Wu et al.^(25)^	Low	Santos-Teles et al.^(36)^	Low
Lasry et al.^(26)^	Low	Crespy et al.^(37)^	Moderate
Genonceaux et al.^(27)^	Low	Katyal et al.^(38)^	Low
Lannes et al.^(28)^	Low	Soliman et al.^(39)^	High
